# Lung microbiome dynamics in health and lung cancer

**DOI:** 10.1099/mgen.0.001509

**Published:** 2025-10-07

**Authors:** Thomas Kanteres, Ismini Maria Angelakopoulou, Eirini Anna Angelakopoulou, Fani Tsolaki, Georgios Tagarakis, Katerina Manika, Maria Exidari, Georgia Gioula

**Affiliations:** 1 Department of Cardiothoracic Surgery, AHEPA University Hospital, Aristotle University of Thessaloniki, Thessaloniki, Greece; 2 Faculty of Medicine, Aristotle University of Thessaloniki, Thessaloniki, Greece; 3 Department of Cardiothoracic Surgery, AHEPA University Hospital, Faculty of Medicine, Aristotle University of Thessaloniki, Thessaloniki, Greece; 4 Department of Pneumonology, Papanikolaou Hospital, Faculty of Medicine, Aristotle University of Thessaloniki, Thessaloniki, Greece

**Keywords:** dysbiosis, lung, lung cancer, microbe–host interaction, microbiome, sequencing

## Abstract

Application of culture-independent sequencing technologies has revealed the presence of a low-biomass, high-diversity microbiome within healthy human lungs. This community is highly dynamic, with microbes constantly being introduced into and cleared from healthy airways, and its multifaceted roles in lung physiology and disease are currently a matter of intensive investigation. In view of the rising lung cancer incidence and mortality rates, a large amount of research has already been conducted in the context of lung cancer, suggesting an association between lung cancer and local dysbiosis. In light of these developments, this review summarizes and discusses existing knowledge on lung microbiome dynamics and composition in health and how these differ in lung cancer patients, focusing on the latest research. Throughout this effort, frequently reported alterations and associated microbe–host interactions in lung cancer development and progression are highlighted, along with some critical methodological considerations, outstanding questions and suggestions for the future.

**Impact Statement**

Human lungs host dynamic microbial communities that are influenced by several intrinsic/extrinsic factors and may affect lung biology and disease in various ways. During the past years, a large number of studies have been performed in the context of lung cancer, revealing several microbiome alterations in lung cancer patients. This review summarizes our current understanding of lung microbiome dynamics in health and lung cancer, focusing on specific lung microbiome alterations that emerge repeatedly throughout the literature and the possible interactions between these microbes and human cells. In addition to bacteria, the latest insights on the emerging roles of fungi and viruses during lung cancer formation/progression are also included. Finally, key methodological considerations that could benefit future endeavours are identified, suggesting the need for a more integrative approach to decode the complex nature of this association and forge new diagnostics and therapies.

## Data Summary

The authors confirm all supporting data, code and protocols have been provided within the article or through supplementary data files.

## Introduction

**Fig. 1.**
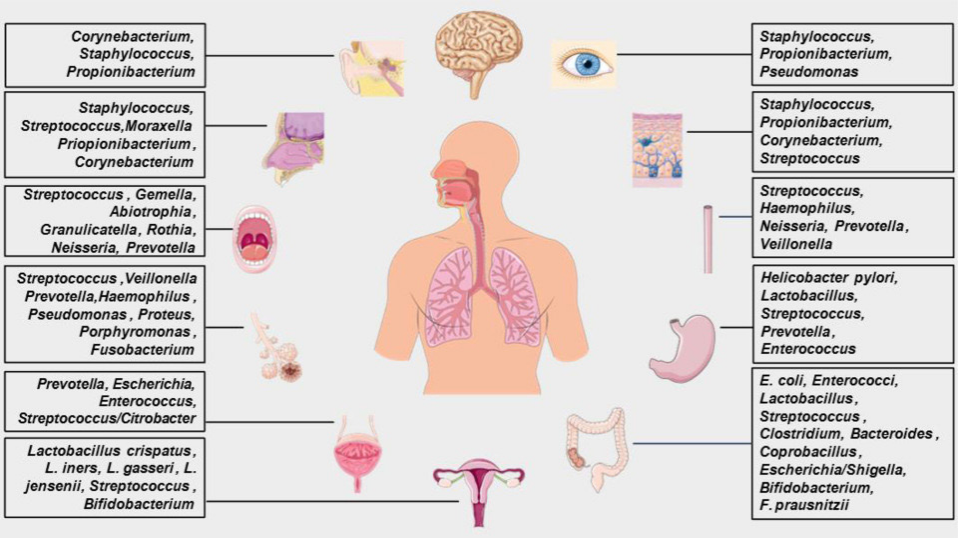
Microbial communities of different biomass and composition are present in various tissues of the human body, influencing several aspects of human physiology either locally or via systemic interactions (e.g. gut microbiome–brain axis). Image created through Servier Medical Art.

The ubiquitous presence of microbes across different tissues and organs of the human body is now widely recognized (Fig. 1). A primary example is the gut, harbouring a highly diverse repertoire of ~10^14^ microbes collectively encoding over 2,000,000 genes (100 times the number of human genes) [1,4]. Symbiosis with such genetically diverse microbial populations generates the substantial mechanistic complexity required for shaping and strengthening the intestinal epithelium, processing a wider range of nutrients, aiding immune defence (e.g. production of bactericidal substances, phages), fine-tuning host immune development and activity, and regulating metabolism, endocrine signalling and nervous system function [5,12]. Other tissues exhibiting extensive colonization with varying microbial compositions and diversities include the skin, oral and nasal cavities and vagina [13,16]. Even though the presence of commensal microbes in these organs has been discussed for a while, tissues with lower concentrations of micro-organisms were long thought to be sterile, mainly due to difficulties in assessing low-abundance microbial communities and culturing certain microbial species. A prominent example is the lower respiratory tract [17], whose microbial content remained unexplored until the development of more sensitive culture-independent DNA sequencing technologies (Table 1). Application of these technologies revealed a low-biomass (2.2×10^3^ bacterial genomes cm^−2^), high-diversity microbiome in human lungs, comprising a combination of bacterial, fungal, archaeal and viral DNA [18,22]. Although DNA sequencing alone cannot differentiate whether the detected DNA derives from living micro-organisms or is extracellular/non-viable, more integrative approaches suggest that the lower respiratory tract, too, likely depends to some extent on microbes for its development, function and immunity. For instance, absence or low abundance of bacteria in mice has been causally linked to altered lung morphology (alveoli significantly reduced and of larger diameter) [23], reduced mucus production [23,24] and a 2.5-fold increase in lung invariant natural killer T cells [25].

**Table 1.** Commonly used culture-independent DNA sequencing approaches to study lung microbiomes

	Microbiome genomic characterization approaches	
	**Amplicon sequencing** (**Short- or long-read**)	**Shotgun metagenomic sequencing**
Summary	**Short-read:** PCR amplification of conserved gene (16S rRNA in bacteria and archaea, 18S rRNA or ITS regions in fungi) and sequencing of the product (hypervariable regions V1–V9 differ across taxa, allowing identification of the micro-organism). Most frequent approach. **Long-read:** Full-length sequencing of the gene, improving resolution. Less common in respiratory research.	Sequencing of *all* the DNA within a given sample. Shotgun reads can then be assembled using a reference genome or *de novo* if a reference genome is not available. Eliminates PCR-introduced bias and allows study of *all* microbial genes. Generates a complex mix of host-derived and micro-organism-derived reads (complicated and expensive for low-biomass samples from human lungs). Suitable for the study of bacteria, fungi and viruses.
Taxonomic resolution	Genus-level (profiling) Species-level (long-read)	Strain-level
Application	Taxonomic analysis Sample diversity Relative abundance	Functional annotation (gene assembly) Inference of function (reference-based analysis) No need for reference genome (*de novo* assembly): identification of yet unknown species (‘dark matter’)
Limitations	Limited throughput PCR biases Dependence on reference genome No functional information	Increased sequencing depth often required Expensive Reference genome information required to infer function

ITS stands for internal transcribed spacer regions.

Accordingly, changes in microbial diversity, composition or relative abundance, commonly referred to as dysbiosis, have been implicated in the initiation and/or progression of various respiratory diseases, including chronic obstructive pulmonary disease (COPD), asthma, bronchiectasis, pneumonia, pulmonary fibrosis and cystic fibrosis [20,26,30]. More recently, intensive microbiome research has expanded towards the field of lung cancer, with many studies suggesting the presence of microecological changes in lung cancer patients. Lung cancer is among the most frequently diagnosed cancers and the leading cause of cancer mortality worldwide, with 2.5 million new cases and 1.8 million deaths (12.4% and 18.7% of all cancer cases and deaths, respectively) estimated in GLOBOCAN 2022 [31]. Under this light, the importance of determining the exact contribution of dysbiosis in lung cancer processes becomes evident. Currently, it is thought that a state of lung and gut dysbiosis may be able to influence lung carcinogenesis and treatment efficacy through various possible mechanisms, including DNA damage and genomic instability, epigenetic dysregulation, immunomodulation disruption, chronic inflammation, metabolism and activation of signalling pathways (thoroughly discussed in the recent reviews by Goto [32], Wang *et al*. [33], Cheng *et al*. [34], Kashyap *et al*. [35] and Li *et al*. [36]). However, the precise roles and possible causality of lung microbiota in lung cancer remain largely elusive. In this regard, identifying possible signatures or other common features within the microbiomes of lung cancer patients via microbial genomics approaches (amplicon sequencing and metagenomics) and studying these mechanistically could offer a more specific view of microbiota effects on lung cancer development and progression, while also improving clinical outcomes by serving various translational roles in patient diagnosis, stratification, monitoring and therapy. The present review discusses the composition and dynamics of lung microbiomes in health and lung cancer, integrating the latest insights from multi-omics studies to explore how the microbiome alterations detected in lung cancer patients may influence specific cancer-relevant processes/pathways. Through this discussion, we aim to highlight new perspectives and more precise avenues to be explored on lung dysbiosis contributions in lung cancer formation/progression. Although most research has so far been conducted on lung bacteria, the latest findings suggest important roles for fungi and viruses in lung function and cancer processes, and these are thus likewise covered in this review. It must be stressed that lung microbiomes may be subject to systemic interactions involving other tissues (i.e. the gut microbiome). Due to the sheer complexity of this rapidly emerging field, however, here we will only focus on lung microbiota in lung physiology and cancer.

## Lung microbial community dynamics

The lower respiratory tract is in constant communication with the external environment, forming an extensive epithelial gas-exchange interface at the level of the alveoli, while it is also anatomically connected to the upper respiratory and gastrointestinal (GI) tracts. As a consequence, microbes can enter the lungs either through bioaerosol inhalation (>10^4^–10^6^ bacteria m^−2^ of atmospheric air) or along with secretions from microbe-laden communicating sites [37,39]. Existing comparisons of bacterial communities obtained via culture-independent approaches (e.g. 16S rRNA sequencing) indicate that lung microbiota mostly derive from the oral microbiome through oropharyngeal aspiration [40,43] and the GI tract through microaspiration of gastro-oesophageal reflux contents [39], while nasal secretions seem to contribute very little to lung microbial populations, at least when not in abnormally high quantities (e.g. as in rhinorrhoea) [43]. Despite the constant provision of micro-organisms, healthy human lungs maintain a low biomass and rich diversity microbial profile, something that is likely important for O_2_/CO_2_ exchange and overall lung function [37,39]. This is possible due to several clearance mechanisms inherent to lung physiology: coughing, mucociliary clearance – whereby motile cilia beating sweeps mucus-trapped micro-organisms and debris towards expectoration/swallowing, the bacteriostatic effects of alveolar surfactants and phagocytosis by alveolar macrophages [44,46]. Despite these mechanisms being currently best understood, it is worth noting that lung selectivity might be more complicated. Interestingly, apart from their role in mucociliary clearance, motile cilia in the established *Euprymna scolopes* squid model have been found to also participate in active symbiont/microbiome recruitment by creating fluid-mechanical microniches that facilitate selection for specific microbial communities [47]. Given the highly conserved nature of cilia across organisms [47], it might be useful to verify whether this selectivity mechanism could also be relevant to other mucociliary surfaces, including the human airway epithelium. In addition to microbial immigration and lung clearance dynamics, lung microecology is also shaped by microbial replication, itself influenced by local conditions (e.g. oxygen availability, access to nutrient sources, pH, temperature and presence of other microbes) [48,49]. Very recent evidence also points to a potentially significant effect of the host’s genetic background in shaping the lung microbiome. In a pioneering study using deep metagenomic sequencing data, Gao *et al*. performed a microbiome–host co-profiling, identifying striking genetic associations between human genes and airway microbes in COPD individuals (e.g. strongest association between the host’s *TBC1D32* intronic SNP rs6917641 and *Streptococcus salivarious*) [50]. Transcriptomic analysis also revealed genetic and transcriptional correlations between the expression of host genes and certain members of the airway microbiome (e.g. *NUDT1*, *MAD1L1* and *Veillonella parvula*, *TTLL9* and *Stenotrophomonas maltophilia* and *LTA4H* and *Haemophilus influenzae*), while Mendelian-randomization analyses pointed to a potential link between *PARK7* expression and the microbial type III secretion system, as well as a genetically mediated association between COPD and increased airway abundance of *Streptococcus intermedius* [50]. Finally, it should be noted that lifestyle and environmental factors are also able to affect lung microbiome composition [51]. A primary example is tobacco smoke, influencing lung microbial communities both directly and indirectly. Smoking can directly introduce microbes into the lungs due to a wealth of microbes and spores often present in tobacco cigarettes [52,53], but also through aspiration of microbes from the oral cavity, itself also modified by tobacco (e.g. enrichment with deleterious and cancer-associated bacteria, such as *Porphyromonas gingivalis* and *Fusobacterium nucleatum*) [54,57]. Regular exposure to tobacco – and nicotine in particular – further influences the lung microbiome by disrupting lung mucociliary clearance mechanisms, as well as by causing immune impairment and chronic inflammation (e.g. affecting innate immune cells, downregulating surface pathogen recognition receptors TLR-2 and MARCO, thereby reducing phagocytic and reactive oxygen species (ROS)-mediated responses against bacteria, and dysregulating B- and T-cells, among others) [58,60]. As a consequence, several studies report significant lung microbiome alterations in tobacco smokers. Indicatively, Liu *et al*. report remarkable changes in the relative abundances ofseveral bacteria in bronchoalveolar lavage fluid (BALF) samples of smokers when compared to those of non-smokers including *Rothia*, *Haemophilus*, *Neisseria*, *Acinetobacter* and *Streptococcus*, among others [61]. The effect of smoking on lung microbiome composition may even be stronger than that of genetics, as highlighted by a twin-family study reporting *Veillonella* and *Megasphaera* enrichment and *Eikenella* and *Haemophilus* reduction due to smoking [62]. Notably, smoking-related disruptions in innate and adaptive immune mechanisms may allow the growth of opportunistic pathogens within lung microbial communities. This might be best illustrated by the enrichment observed in the lungs of smokers with the biofilm-forming and multi-drug-resistant bacteria *Serratia marcescens* and *S. maltophilia*, an effect that correlated with smoking intensity and duration [63]. In summary, lung microbial landscapes exist in a dynamic state of flux, in which a variety of microbes are constantly introduced and cleared to preserve physiological homeostasis, and the resulting overall composition is affected by several intrinsic and extrinsic factors.

## Healthy lung microbiomes

Culture-independent sequencing analyses suggest significant inter-individual variability in the lung microbiome composition of healthy adults. Nonetheless, certain microbes have consistently been reported across studies. Among bacteria, Firmicutes (currently termed Bacillota) and Bacteroidetes (currently termed Bacteroidota) seem to be the most common phyla in healthy lungs, and of these, the oral commensals *Streptococcus*, *Veillonella* and *Prevotella* seem to be the dominant genera [19,20, 29, 64,66]. Other bacteria that are also commonly detected in healthy lungs include the Proteobacteria (currently termed Pseudomonadota) *Haemophilus*, *Pseudomonas* and *Proteus*, but also *Porphyromonas* (Bacteroidota) and *Fusobacterium* (Fusobacteriota) [19,20, 64]. However, it should be noted that these might not be the case for all individuals, as 16S rRNA sequencing analysis of BALF samples from asymptomatic individuals by Segal *et al*. revealed two pneumotypes; half of the individuals examined harboured a low abundance of background/environmental predominant taxa (similar to the bronchoscopy saline solution), and the remaining half presented enrichment in oral taxa (*Prevotella*, *Veillonella* and *Streptococcus*) [29]. Interestingly, the latter pneumotype was also associated with increased subclinical lung inflammation [29]. Concerning the topographical distribution of bacteria in healthy individuals, intra-individual variability in composition across different locations of the airways does exist; this is, however, less pronounced than inter-individual variability and mostly depends on the distance of a given site from the oral cavity, microbial immigration and lung clearance mechanisms [37,48]. From a functional standpoint, the exact roles of these bacteria in lung physiology remain largely unclear. This is partly due to the use of 16S rRNA gene sequencing in most studies – a methodology that circumvents issues previously seen with traditional culturing approaches nonetheless presents its own limitations. Some of these include its inability to differentiate between intracellular and extracellular DNA, limited taxonomy resolution (up to genera and species level but no information on strains, yet different strains of the same species often exhibit marked differences in their genes), lack of functional information, and bias issues such as non-linear amplification, lack of primers for certain micro-organisms and dependence on reference databases of already annotated microbes [67,69]. These limitations reflect the need for more powerful DNA sequencing methods (e.g. shotgun metagenomics, whole-genome sequencing) and the use of integrative (multi-omic) approaches combining these with techniques providing a more direct assessment of microbial viability and functional processes (metatranscriptomics, metabolomics, culturomics, etc.) [68,70]. Nonetheless, existing integrative studies indicate that the lung microbiome is indeed alive and metabolically active [68]. Although it might be participating in various aspects of lung physiology, most research to date suggests it plays important roles in lung immunity, including the regulation of innate and adaptive immunity [71,75].

Less is known about the fungal and viral members of lung microbial communities in health, mostly due to practical difficulties in assessing the mycobiome (e.g. difficulty in extracting fungal DNA due to lysis-resistant cell walls, presence of DNA-mimicking compounds, biases in 18S rRNA amplification) and virome (absence of conserved genes) [76,77]. Existing studies, however, indicate the presence of a low-biomass fungal population in the healthy lung, including the Ascomycota and Basidiomycota phyla and the genera *Candida*, *Saccharomyces*, *Penicillium*, *Cladosporium* and *Fusarium* [21,22, 78,80]. Although the role of fungi on lung physiology currently remains unclear, the existence of a mycobiome–bacteriome equilibrium has been discussed in the gut following the discovery of significant fungal expansions in mouse models during antibiotic treatment, a finding that was reversed after antibiotic discontinuation [81]. Using network analysis, Huang *et al*. indicate that fungi–bacteria interkingdom correlations exist also in the healthy human airways and that these connections are decreased in untreated asthma and inhaled corticosteroid-treated patients [22]. Further studies are, however, required to interpret these correlations. Finally, metagenomics approaches have revealed the existence of a low-complexity respiratory virome in healthy lungs, composed mostly of phages with some eukaryotic viruses [18,82]. Among eukaryotic viruses, Picornaviruses, Paramyxoviruses, Orthomyxoviruses, Coronaviruses, Adenoviruses, Parvoviruses, Herpesviruses, Anelloviruses, Papillomaviruses and Polyomaviruses have all been detected in the human respiratory tract (reviewed by Wylie) [83]. Healthy viromes exhibit extensive variability both in terms of intra-individual regional composition across the airways and overall composition between individuals [84,85], yet *Anelloviridae* seem to be the most common eukaryotic viruses in the human respiratory tract, followed by the recently discovered family of *Redondoviridae* [80,86]. Although their exact roles in health and disease remain largely elusive [80], these seem to correlate with immune system function and bloom during disease [80,86, 87].

## Microbiome alterations in lung cancer

Lung cancer tumours are subdivided into two major histological types: non-small cell lung carcinoma (NSCLC) and small cell lung carcinoma (SCLC), of which NSCLC is by far the most frequent lung cancer diagnosis (80–85% of all cases) [88]. NSCLC is further classified into lung adenocarcinoma (LUAD) (~40%), lung squamous cell carcinoma (LUSC) (~30%), large-cell carcinoma (10–15%) and rare NSCLC subtypes, including adenosquamous carcinoma, pleomorphic sarcomatoid carcinoma, large-cell neuroendocrine carcinoma and carcinoid tumour [88]. Research into the molecular basis of lung cancer over the past several years has revealed a range of genetic drivers (point mutations, amplifications, rearrangements and gene expression alterations), such as the *EGFR*, *KRAS*, *FGFR1* and *PTEN* gene mutations frequently encountered in NSCLC [88]. In addition to endogenous factors, the impact of external influences (e.g. tobacco smoking/secondhand smoke, air pollution and occupational exposure to chemicals, asbestos and radon gas), chronic lung disease (e.g. COPD) and certain infections (e.g. *Mycobacterium tuberculosis*) on increased lung cancer risk has long been discussed [89,90].

More recently, numerous studies have begun to uncover several microbiome alterations in lung cancer patients, suggesting that lung dysbiosis may also influence lung cancer initiation/progression and clinical outcomes. The biological significance of these changes and their potential roles in these processes, however, remain unclear. This uncertainty is further compounded by inconsistent and often conflicting findings regarding the specific taxa, relative abundances and ecological diversities reported across studies. These inconsistencies likely reflect the biological complexity of human lung microbiomes, which are characterized by inherent inter-individual variability and a largely enigmatic functional capacity (e.g. different microbes may have similar effects on the lung microenvironment, yet the same microbe may play opposite roles in different contexts), as well as study-specific factors (e.g. small sample sizes, controls often involving other pathologies and sampling/analytical differences). Despite these variabilities, however, several microbial alterations consistently emerge across different cohorts and study designs, reflecting possible functional involvement in lung cancer. This section discusses lung microbiome alterations that have been detected in lung cancer patients and the potential mechanisms linking these to lung cancer processes, highlighting, whenever possible, some of the most recurrent findings (Table 2).

**Table 2.** Lung microbiome alterations detected in lung cancer patients across studies reviewed here

Source	Sampling	Analysis	Taxa	Alteration
Lower airways and lungs	Sputum	Metagenomic sequencing	*G. adjacens*, *Streptococcus* sp. (various)	Abundance sign. increased in patients. *G. adiacens* was more common in non-smokers, associated with several *Streptococcus* spp. in patients but not controls, titre related to disease status [119,120]
	Protected bronchial specimen brushing	16S rRNA sequencing	*Streptococcus*	Enriched in patients [65]
	Airway brushing	16S rRNA and paired transcriptome sequencing	*Streptococcus*, *Prevotella*, *Veillonella*	Enrichment of supraglottic-predominant taxa in patients. Enrichment associated with ERK1/2 and PI3K signalling pathway upregulation *in vivo* and *in vitro* [66]
	BALF	16S rRNA sequencing	*Streptococcus*	Enriched in patients [102]
		16S rRNA sequencing	*Veillonella*, *Megasphaera*	Increased abundance in patients when compared to individuals with benign mass lesions [122]
		16S rRNA sequencing, culturomics	*Str. intermedius*, *Ge. sanguinis*	*Streptococcus* and *Veillonella* highly dominant in patients. *Str. intermedius* and *Ge. sanguinis* were more abundant in the NSCLC compared to SCLC [121]
		Metagenomic sequencing	*Str. mitis*	Highest value for differentiating cancer from benign diseases. Increased from benign disease to adenocarcinoma *in situ*, to minimally invasive adenocarcinoma, to invasive adenocarcinoma [97]
		Metagenomic sequencing	*Prevotella*, *Streptococcus*, *Veillonella*, *Rothia*, *Capnocytophaga*	Significantly higher mapping read number in patients. *Veillonella* and *Prevotella* were more prevalent in smokers, males, LUSC, FEV1/FVC ≥70%, TNM I-II (*Veillonella*) and III-IV (*Prevotella*). *Streptococcus*, *Rothia* and *Capnocytophaga* more prevalent in non-smokers, females, LUAD, FEV1/FVC <70%, TNM III-IV [123]
	BALF (lobectomy)	Metagenomic sequencing	*C. globosum*	Decreased in NSCLC patients [91]
Intra-tumoural	Tumour tissue	16S rRNA sequencing	*Acidovorax*	More frequent in LUSC smokers with TP53 mutations. Gene–microbiome-smoking association may be promoting tumourigenesis [99]
		16S rRNA sequencing	*Cyanobacteria*	Increased in LUAD. Cyanobacteria microcystin correlated with CD36 reduction, PARP1 increase. CD36 may internalize microcystin increasing the levels of PARP1 [127]
		16S rRNA sequencing, metabolomics	*Ralstonia Akkermansia*, *Escherichia-shigella*, *Klebsiella*	*Ralstonia* enriched in early LUAD. *Akkermansia*, *Escherichia-shigella* and *Klebsiella* enriched in early LUSC. Early LUAD mostly relevant to energy metabolism (microbiota likely functioning via *N*-acetyl-1-aspartylglutamic acid). Early LUSC was mostly relevant to glutathione metabolism and redox biology (microbiota likely functioning through creatine and *N*-acetylmethionine) [130]
		Metagenomics, transcriptomics, proteomics	*M. discipulorum*	Enriched in early LUAD, but nearly absent in healthy controls. Microbiota–host interplay (e.g. microbiota abundance alterations correlated with regulation of ribosomal and histone genes, *M. discipulorum* and specific microbiota-related histone modifications correlated to and likely modulate the relationship of MUC1 and ST6GALNAC1, *M. discipulorum* correlated to GOLM1) [131]
		16S rRNA sequencing	*Thermus*, *Legionella*	*Thermus* abundance correlates to advanced disease (TNM IIIB, IV). *Legionella* is more abundant in patients with metastases [126]
		ITS2 seq, metagenomics (TCGA), stainings	*Aspergillus*, *Agaricomycetes*	Enriched in lung tumours of current smokers compared to never smokers [138]
		Metagenomic analysis of WGS data (TCGA)	*B*. *dermitidis*/*gilchristii*	Detected in 6/50 LUSC samples compared to general incidence of 1-2/100,000 [139,140]
		Metagenomic sequencing	*A. sydowii*	Enriched in LUAD and this was associated with immunosuppression and poor disease outcome. *A. sydowii* promoted tumour progression via the *β*-glucan/Dectin-1/CARD9 pathway and subsequent macrophage IL-1*β* secretion, inducing the expansion and activation of myeloid-derived suppressor cells, and resulting in cytotoxic T lymphocyte suppression and PD-1+CD8+T cell accumulation in murine lung cancer models [141]
		Metagenomics, metatranscriptomics	Several, including *Sa. paradoxus*, *Y. lipolytica*	*Sa. paradoxus* decreased and *Y. lipolytica* increased in patients. Altered fungal abundances associated with histone, ribosomal protein and NADH–ubiquinone oxidoreductase pathway genes in early LUAD. Mycobiome–host TIICs interplay (e.g. *Sa. paradoxus* associated with changes in neutrophils and Tregs, *Y. lipolytica* with resting dendritic cells). Mycobiome–host interplay may influence OS (e.g. *Sa. paradoxus* correlated to host TFs and associated with prognostic gene signature) [142]
		Metagenomic sequencing	*Al. arborescens*	Higher abundance in NSCLC [143]
		Metagenomics, metabolomics	*T. marneffei*	Most significantly differential fungus between NSCLC patients and controls. Enrichment promoted lung cancer cell growth by triggering M2 macrophage polarization through activation of the arginine-ornithine cycle [153]

Note: Existing information on viruses (HPV, MCPyV, EBV and JCV) derives from targeted analyses, and as such, it is not directly comparable to taxa detected through sequencing-based lung microbiome interrogations.

## Bacteria

Conflicting results have been reported regarding alpha-diversity (abundance of different species within one habitat/sample) and beta-diversity (variability between different samples) in lung cancer. Many studies have found decreased alpha-diversity in lung cancer (either when comparing patients to healthy/benign disease controls or tumours to paired non-tumour segments) in different sample types [65,91,98], although some studies finding it increased [99,103] or unaltered [104] also exist. Moreover, using protected bronchial specimen brushing samples, Liu *et al*. reported a decline in alpha-diversity from healthy to non-cancerous to paired cancerous sites, suggesting changes at the local microenvironment taking place along with lung cancer development [65]. Changes in beta-diversity have also been reported, with some studies detecting an increase [105,106] and others a decrease [94,104, 107]. In a meta-analysis of four studies comparing the microbiome in lung tumour tissues against tumour-adjacent normal tissues, Najafi *et al*. found a decrease in alpha-diversity in lung cancer, but no significant difference between the two groups in terms of beta-diversity [108]. According to a very recent analysis of spontaneous sputum samples, airway microbiota diversity may be associated with systemic inflammation in lung cancer, as evidenced by the loss of alpha-diversity in NSCLC patients with relatively high inflammation status and the differences in microbial structure between patients with relatively high inflammation status and those with low inflammation status [109]. Other recent studies found poorly differentiated NSCLC to be associated with a lower microbial diversity [110], and that lower tumour microbiome diversity significantly correlates to reduced recurrence-free survival of lung cancer patients [111]. Although diversity alone is not an absolute index of a microbiome’s health state [112], these studies suggest that lung microecological diversity is frequently altered in lung cancer samples compared to controls – mostly decreased – and that this correlates with significant biological and clinical parameters.

In addition to alterations in ecological diversity, changes in the abundance of certain bacteria have also been frequently reported in lung cancer patients. Using 16S rRNA gene amplicon sequencing and paired transcriptome sequencing on *airway brushing samples*, Tsay *et al*. revealed an overall enrichment of supraglottic-predominant taxa such as *Streptococcus*, *Prevotella* and especially *Veillonella* in the lower airways of lung cancer patients when compared to controls [66]. Enrichment with these taxa was associated with upregulation of the ERK1/2 and PI3K signalling pathways *in vivo*, but also *in vitro* upon exposure of an immortalized epithelial cell line to these micro-organisms [66]. This effect may be attributed to the interaction of microbe-derived immune-active molecules with host epithelial cells via pattern recognition receptors [66]. Importantly, both the ERK and PI3K signalling pathways are critical regulators of survival, proliferation, differentiation and motility of the cell in response to extracellular cues [113], and dysregulation of these pathways has been previously detected in lung cancer. A few examples include dysregulation of PI3K/Akt activation early in lung cancer [114] and its correlation to NSCLC high grade and advanced disease [115], as well as the correlation of ERK1/2 activation with advanced and aggressive NSCLC tumours [116]. In 2021, Li *et al*. also reported evidence of these pathways’ participation in the maintenance of lung cancer stem-like cell (CSC) self-renewal [117]. Later, Li *et al*. found that *Streptococcus pneumoniae* invades lung cancer cells by binding to the platelet-activating factor receptor (PAFR), which in turn activates the PI3K/AKT and NF*κ*B signalling pathways and pro-inflammatory responses promoting lung cancer development and progression [118]. Apart from Tsay *et al*., several other studies have also confirmed *Streptococcus* enrichment in lung cancer patients using different lower airway sampling methods and genomic characterization approaches. For instance, 16S rRNA amplicon sequencing analysis of *protected bronchial specimen brushing* samples also detected *Streptococcus* enrichment in lung cancer patients when compared to controls [65], while metagenomic analyses of *sputum* samples showed a significant increase in the abundance of *Granulicatella adiacens* and various *Streptococcus* species (among others) in lung cancer patients when compared to controls [119,120]. *G. adiacens* was more common in non-smokers’ samples, associated with several *Streptococcus* species in cancer patients but not in controls, and its titre correlated to disease status [120]. Using *BALF* samples, Kim *et al*. found *Streptococcus* to be the most abundant genus in patients with lung cancer and to be significantly enriched in lung cancer patients compared to controls [102], while Yuan *et al*. identified *Streptococcus mitis* as the species with the highest diagnostic value for differentiating lung cancer from benign lung diseases through metagenomics [97]. Interestingly, *Str. mitis* (along with two *Burkholderia* species) also displayed an increasing trend from benign lung disease to adenocarcinoma *in situ*, minimally invasive adenocarcinoma and invasive adenocarcinoma groups, suggesting changes along with adenocarcinoma development [97]. Another pioneering study combining culturomics and 16S rRNA gene amplicon sequencing also identified *Streptococcus* and *Veillonella* as highly dominant in *BALF and oral* samples from lung cancer patients, highlighting the importance of orally introduced lung dysbiosis in lung cancer [121]. In the same study, *Str. intermedius* and *Gemella sanguinis* were isolated only from the NSCLC group, and these were found to be more abundant in the NSCLC when compared to the SCLC group by 16S rRNA gene sequencing [121]. The addition of culturomics here was a significant improvement, as it allowed isolation of *living* bacteria and achievement of strain-level resolution, two major caveats of 16S rRNA sequencing. Increased abundance of *Veillonella* and *Megasphaera*, both members of the Veillonellaceae family, has also been detected in *BALF* samples from lung cancer patients when compared to individuals with benign mass lesions [122], a finding that is consistent with the significant association detected between *Veillonella*, *Megasphaera* and upregulation of cancer-relevant pathways in *airway brushing* samples by Tsay *et al*. [66]. In a very recent metagenomics analysis of *BALF* samples, *Prevotella*, *Streptococcus*, *Veillonella*, *Rothia* and *Capnocytophaga* were found to be the most prevalent genera among the lower respiratory tracts of lung cancer patients and showed a significantly higher mapping read number in cancer patients when compared to non-cancer controls [123]. Upon stratification of patients in subgroups according to smoking status, gender, tumour histology, pulmonary function and TNM stage, *Veillonella* and *Prevotella* emerged as more prevalent among smokers, males, LUSC patients and those with forced expiratory volume in 1 s/forced vital capacity (FEV1/FVC) ≥70%, with *Veillonella* predominating in TNM stages I-II and *Prevotella* in stages III-IV, while *Streptococcus*, *Rothia* and *Capnocytophaga* were more prevalent in non-smokers, females, LUAD patients, cases with FEV1/FVC <70% and TNM stages III-IV [123]. Interestingly, these microbial prevalence patterns according to LUSC/LUAD tumour histology have also been detected in other studies. For instance, Yan *et al*. have found the levels of *Veillonella* and *Capnocytophaga* to be significantly higher in *saliva* samples from lung cancer patients, with *Veillonella* serving as a better biomarker of LUSC and *Capnocytophaga* for LUAD [124]. Along the same lines, Leng *et al*. have suggested *Veillonella* along with *Acidovorax* as promising *sputum* biomarkers for LUSC and *Capnocytophaga* for LUAD [125]. These consistent patterns across different sample types and patient cohorts suggest that orally introduced *Veillonella* and *Capnocytophaga* could somehow be relevant to LUSC and LUAD processes, respectively, underscoring the importance of patient stratification when interrogating the microbiota of lung cancer patients, as well as the need to assess the role(s) of these bacteria in the relevant histological subtype. Delving deeper into the possible roles of these five genera in lung cancer, gene set variation analysis on transcriptomic data from 987 TCGA-NSCLC samples revealed significant positive correlations between these genera and various cancer-relevant biological processes [123]. These included the ‘TCA cycle and glycolysis/gluconeogenesis’ consistently with the Warburg effect and tumour metabolic reprogramming, ‘DNA replication and nucleotide excision repair’ and ‘p53 signalling’ pathways, suggesting possible roles in tumour cell survival, proliferation and DNA damage response/genomic instability, as well as the oncogenic ‘PI3K-Akt, Notch and Hippo pathways’, known to promote cell growth, proliferation and survival and often found dysregulated in cancers leading to uncontrolled cell growth, resistance to apoptosis and metastasis [123]. Other terms included ‘T/B cell receptor signalling’ and ‘IL-17’, suggesting possible involvement of these taxa in immune mechanisms, such as inflammation or immune evasion, as well as ‘platinum drug resistance’, ‘pathways in cancer’ and ‘small cell lung cancer’, pointing to a possible involvement in chemotherapy resistance and signalling pathways likely shared by NSCLC and SCLC [123]. Although these findings cannot prove causality, taken together, these lower airway analyses suggest that enrichment with certain orally introduced bacteria is a frequent finding in lung cancer and that these bacteria may interact with oncogenic programmes controlling tumour progression, immune modulation and therapeutic response. It might thus be useful to further investigate these bacteria and associated pathways in appropriate experimental contexts in lung cancer.

Several *tumour tissue*-based studies, on the other hand, mostly point to significant Proteobacteria presence in lung cancer patients [95,98, 99, 126, 127], possibly with the exception of Halomonadaceae and *Halomonas* [128]. More conflicting findings have been reported about Firmicutes and Actinobacteria (Actinomycetota) in lung biopsy studies, yet in their systematic review and meta-analysis of four lung cancer tissue-based studies, Najafi *et al*. found a significant decrease in Actinobacteria but no decrease in Firmicutes (with the exception of *Lachnoanaerobaculum*) [128]. With regard to histological subtypes, Greathouse *et al*. found the proteobacterium *Acidovorax* to be more frequent in LUSC smokers with TP53 mutations, highlighting a gene–microbiome association whereby a smoke-related bacterium could be taking advantage of a smoking/mutation-inflicted epithelial microenvironment to promote tumourigenesis [99]. In a cross-sectional study, Apopa *et al*. found Cyanobacteria to be specifically increased in LUAD tissue samples and that the Cyanobacteria-derived toxin microcystin correlates with a reduction in the levels of CD36 (a receptor of acute inflammation known to internalize microbes and toxins) and an increase in the levels of the cell proliferation regulator PARP1 [127]. This association, shown through *in silico* pathway analysis and confirmed in both microcystin-treated NSCLC cell lines and Cyanobacteria-positive LUAD samples, suggests that CD36 may internalize microcystin, increasing the levels of PARP1 [127]. Interestingly, PARP1‐mediated AMPK‐mTOR signalling plays an important role in the proliferation, migration and invasion of lung cancer cells [129]. More recently, several tissue-based studies have focused on the role of lung microbiota in early-stage lung cancer, often using integrative/multi-omics approaches to study distinct histological subtypes. For instance, a latest study using 16S rRNA sequencing on a large number of surgically excised tissue samples found the proteobacterium *Ralstonia* to be significantly enriched in early LUAD samples, likely suggesting a role for this bacterium in early LUAD development, while *Akkermansia* (Verrucomicrobiota), *Escherichia-shigella* (Proteobacteria) and *Klebsiella* (Proteobacteria) were more abundant in early LUSC [130]. Non-targeted metabolomics and correlation analysis further revealed that early LUAD differential metabolites were mostly relevant to energy metabolism (with differential microbiota likely functioning via *N*-acetyl-1-aspartylglutamic acid), while early LUSC differential metabolites were mostly relevant to glutathione metabolism and redox biology (with differential microbiota likely functioning through creatine and *N*-acetylmethionine) [130]. Another recent study of early-stage LUAD specimens integrating ultra-deep metagenomics, transcriptomics and proteomics not only characterized local dysbiosis in early LUAD samples (e.g. enrichment of the proteobacterium *Methyloversatilis discipulorum*) when compared to healthy controls (pneumothorax adjacent tissues) but went further to reveal the presence of a complex interplay between these microbes and host biology [131]. Indicatively, significant correlations were observed between specific microbiota abundance alterations and the regulation of ribosomal and histone genes, as well as potential interactions between microbial communities and histone modifications [131]. This finding is crucial, as it suggests that local dysbiosis can instigate tumourigenic alterations at both the transcription and translation levels in the host, contributing to the aetiology of LUAD carcinogenesis [131]. Furthermore, *M. discipulorum* and specific microbiota-related histone modifications were found to correlate to and likely modulate the relationship between MUC1 and ST6GALNAC1 [131]. Interestingly, MUC1 is a mucin glycoprotein and well-known oncogene often aberrantly expressed in LUADs [132], while ST6GALNAC1 is a mucin‐type *O*‐glycosyltransferase known to promote cancer progression by altering the *O*‐glycosylation pattern of glycoproteins [133]. Of note, another latest study employing 16S rRNA sequencing and lectin microarrays also found a correlation between altered protein glycopatterns and lung dysbiosis in LUAD tissues, whereby glycoproteins with multivalent Sia and (GlcNAc)n structures significantly reduced the adhesion and toxicity of the proteobacterium *Sphingomonas mucosissima* to lung cancer cells [134]. These findings suggest that a complex and possibly bi-directional relationship may exist between microbes and protein glycosylation patterns in the lungs of LUAD patients. Further investigation of this relationship and its role in LUAD carcinogenesis/progression is thus warranted. Apart from this, transcriptomic and proteomic analyses by Sun *et al*. showed a correlation between *M. discipulorum* and GOLM1 [131], an epithelial Golgi transmembrane protein known to promote growth and metastasis in various cancers [135]. Previous studies have also suggested an association between GOLM1 overexpression and LUAD [135,137], something that could be relevant to the *M. discipulorum*–GOLM1 correlation found by Sun *et al*. and provide another link to explore in LUAD dysbiosis. Regarding later lung cancer stages, Yu *et al*. identified that higher *Thermus* abundance correlates to advanced disease stage (IIIB, IV) and that *Legionella* is more abundant in patients who develop metastases, suggesting that these bacteria might be somehow related to disease progression [126]. Although these tumour tissue-based studies mostly highlight Proteobacteria, it should be noted that detection of oral taxa (e.g. *Streptococcus*) in the lower airways might not be solely attributed to bronchoscopy contamination, as these were also present in lobectomy-derived BALF [91], suggesting possible introduction into the lungs by microaspiration and that inconsistency with tissue-based results could be – at least in part – due to other factors. In this regard, these inconsistencies could also be reflecting differences between lower airway and intra-tumoural microbial communities. BALF, for instance, collects a complex mixture of cells, mucus and soluble luminal microbiota, more likely reflecting oral/upper airway dispersion, while tissue-based studies often involve formalin-fixed paraffin-embedded or fresh frozen tumour samples, directly accessing microbes present within the tumour microenvironment. Intra-tumoural communities often survive under hypoxia, altered metabolism, oxidative stress and other tumour-associated conditions and may have a different profile than lower airway microbes. Different microbial alterations may therefore be valid, influencing lung cancer processes and the lung microenvironment in general, through different ways/routes. Confirmation of these findings in larger cohorts and mechanistic studies for all of these frequently highlighted microbes in the right context may thus be required to obtain a better understanding of microbiota contributions in lung cancer formation and progression.

## Fungi and viruses

The role of the mycobiome and virome in lung cancer is currently understudied. Nonetheless, existing pan-cancer mycobiome studies (including lung cancer) indicate that fungi are generally low in abundance, constituting a smaller proportion of the microbiome than bacteria, and are often intracellular in terms of localization, while fungal load increases in cancer, exhibiting cancer-type-specific richness, composition and localization patterns [138,139]. Specifically with regard to lung cancer, several characteristics emerged, among which were a significantly higher fungal load in tumours when compared to adjacent tissue, higher intra-tumoural richness, a macrophage localization pattern and significant positive correlations between intra-tumoural fungal and bacterial richness [138]. Moreover, current smokers exhibited enrichment in *Aspergillus* (Ascomycota) and *Agaricomycetes* (Basidiomycota) when compared to never smokers [138]. In another pan-cancer study, *Blastomyces* (*Blastomyces dermitidis/gilchristii*) (Ascomycota) was found in the tissues of 6/50 LUSC patients [139]. Considering a general incidence of 1-2/100,000 [139,140], this association is evidently striking, and further investigation is required to decipher the exact role of this fungus in LUSC pathogenesis. In LUAD patients, Liu *et al*. identified intra-tumoural enrichment of *Aspergillus sydowii* using deep shotgun metagenomic sequencing, a finding that was associated with immunosuppression and poor disease outcome [141]. Using three syngeneic murine lung cancer models, the authors further demonstrated that *A. sydowii* promotes tumour progression via the *β*-glucan/Dectin-1/CARD9 pathway and subsequent macrophage IL-1*β* secretion, which in turn induces the expansion and activation of myeloid-derived suppressor cells, resulting in cytotoxic T lymphocyte suppression and PD-1+ CD8+ T cell accumulation [141]. More recently, a multi-omics study combining ultra-deep metagenomics and metatranscriptomics has characterized the tumour-resident mycobiome in early-stage LUAD patients with no smoking history, revealing the existence of mycobiome–host interactions in these patients [142]. More specifically, reduced species diversity, altered abundances in specific fungi (including some belonging to the *Aspergillus* genus) and weak fungal–bacterial correlations were observed in early LUAD patients when compared to non-cancer controls, suggesting that changes in the structure of the mycobiome occur already from the earlier stages of LUAD development and that imbalances in certain fungi and in interspecies correlations may be relevant to LUAD carcinogenesis [142]. Associations between certain fungi enrichments and specific clinicopathological features were also observed, suggesting possible involvement of these fungi in the mechanisms determining the clinicopathological features and progression of early-stage LUAD (e.g. Saccharomycetes and Saccharomycetales were enriched in minimally invasive adenocarcinoma, *Heterobasidion irregulare* in invasive adenocarcinoma and in patients with solid nodules, *Aspergillus pseudonomius* in patients with pure ground glass nodules and several *Aspergillus* species in early multiple primary lung cancer) [142]. Microbial functional analysis revealed significant alterations associated with histone, ribosomal protein and NADH–ubiquinone oxidoreductase pathway genes, indicating that alterations in microbial functional genes and synthetic metabolic pathways are likely involved in early LUAD [142]. A complex interplay between fungi and host tumour-infiltrating immune cells (TIICs) was also observed in these patients (e.g. *Saccharomyces paradoxus* associated with changes in neutrophils and Tregs and *Yarrowia lipolytica* with resting dendritic cells, among others), suggesting these fungi may influence the tumour immune microenvironment and tumour progression. Finally, *Sa. paradoxus* also correlated to a range of host transcription factors and was strongly associated with a signature of three prognostic genes (*GRIA1*, *CDO1*, *FHL1*), suggesting that the mycobiome–host interplay may ultimately influence overall survival (OS) [142]. Apart from these studies highlighting the involvement of *Aspergillus* and Saccharomycetes fungi in LUAD and *Blastomyces* in LUSC, other studies have mostly studied NSCLC groups. In their recent metagenomics study, Zhao *et al*. reported increased mycobiota alpha-diversity and a higher abundance of the fungus *Alternaria arborescens* (Ascomycota) in NSCLC, both of which positively correlated with disease progression [143], while Zheng *et al*. reported depletion of *Chaetomium globosum* (Ascomycota) in NSCLC patients [91]. Interestingly, *C. globosum* is not a known member of the human microbiome. It is instead a saprophytic fungus commonly found on decaying matter and an opportunistic pathogen known to cause allergic reactions through a range of mycotoxins it produces, nail and skin infections, and more rarely, severe infections like brain abscesses, pneumonia and endocarditis, particularly in immunocompromised individuals. Despite its adverse effects on mammalian cells, however, it has lately become a matter of intensive research due to the antitumor and antimicrobial properties of several of its compounds, such as the cytochalasins chaetoglobosins A-G, chetomin, chaetomugilides A-C and chaetoviridins [144,145]. The mycotoxins chaetoglobosins are long known to inhibit cell movement and cytoplasmic division [146] and have been found to inhibit the growth or induce cytotoxicity in various human cancer cell lines (breast, colon, gastric, prostate, hepatocarcinoma) [145,147, 148], while armochaetoglobosin C showed strong antimicrobial activity against *Klebsiella pneumoniae* [145,149]. Chetomin and chaetoviridins J and K have also been found to exert tumour growth inhibitory and cancer chemo-preventive actions via hypoxia-inducible transcription blockage [150] and TNF-*α*-induced NF*κ*B inhibition [151], respectively. Especially with regard to NSCLC, chetomin has been found to effectively target both CSCs and non-CSCs by inhibiting the binding of Hsp90 to its downstream effector hypoxia-inducible factor 1 alpha [152]. These effects were detected both *in vitro* (inhibited CSC sphere-forming within a nanomolar range and non-CSC proliferation within a micromolar range) and *in vivo* (decreased tumour formation in several NSCLC mouse models) without observable toxicity to the animals [152]. Considering that the adverse health effects of this fungus are generally thought to depend on its growth and the concentration of its byproducts, it might be interesting to verify whether other studies also detect this fungus in human lungs in the absence of infection and, if yes, its possible effects in such a low-biomass context. By extension, it could also be worthy to explore whether lack of *C. globosum* products would indeed somehow influence lung cancer formation/progression *in vivo* (either through these compounds’ direct inhibitory/cytotoxic effects on lung cancer cells or their antimicrobial activities controlling the growth of other members of the lung microbiome with known carcinogenic effects). In addition to these studies, a very recent integrative study combining shotgun metagenomics, metabolomics and *in vitro/in vivo* assays identified the fungus *Talaromyces marneffei* (Ascomycota) as the most significantly differential fungus between NSCLC patients and non-cancer controls and found that enrichment with this fungus was able to promote lung cancer cell growth by triggering M2 macrophage polarization through activation of the arginine-ornithine cycle [153].

Lastly, several viruses have been detected in lung tumours (mostly via PCR and *in situ* DNA hybridization), but whether these are causative agents in lung cancer development or the exact role of these viruses in lung cancer remains largely unclear. Some of these include human papillomavirus (HPV), Merkel cell polyomavirus (MCPyV), Epstein–Barr virus (EBV) and John Cunningham virus (JCV) [154], a list that is likely to expand with increasing use of shotgun sequencing. HPV infection rates (and especially HPV 16, HPV 18) seem to be higher among lung cancer tissues (HPV 16: 19.8%, HPV 18: 18.59%) when compared to controls (HPV 16: 5.84%, HPV 18: 4.29%) [154,155], while among different histological subtypes, HPV infection rates seem to be more prevalent in LUSC (25.1%) when compared to LUAD (15.1%) [154], probably due to a greater affinity of the virus with squamous epithelial cells [155]. Another study found MCPyV DNA in 35.7% of the LUAD and in 40.7% of the LUSC samples examined, while also finding an association of the virus with disease progression, with MCPyV LT-Ag being higher in stage IV (0.204×10^−3^ copies cell^−1^) than in any other stage [156]. EBV, on the other hand, seems to be more common among patients with pulmonary lymphoepithelioma-like carcinoma in east/south-east Asian populations [154]. Another example is the study of JCV in 103 lung carcinomas and 18 normal lung tissues by Zheng *et al*., where a higher JCV detection rate and copy number were observed in lung cancer when compared to normal lung tissues, and high JCV copies were associated with increased proliferation and downregulation of cell adhesion [157]. Many studies have been conducted for all these viruses, suggesting several possible oncogenic mechanisms (reviewed in detail by Hu *et al*.) [154]. However, the small percentage of infected individuals developing lung cancer, long latency periods hindering the study of viral causality and indirect carcinogenic effects of some viral agents complicate our understanding of the mechanisms by which viruses may be contributing to cancer (e.g. chronic infection and inflammation, sustaining tumour cells via viral products or directly contributing to cancer cell transformation by viral oncogenes) [154]. Further research and technology/model development are likely required to answer these questions.

## Important modulating factors

It is important to note that several modulating factors may influence the lung microbiome profiles detected in lung cancer patients. Among these, tobacco smoking has emerged as a major and independent modifier of lung microbial communities. Its disruptive effects on mucociliary clearance, immune responses and chronic inflammatory mechanisms result in considerable microbial shifts that often resemble those detected in lung cancer patients. For instance, enrichments with *Streptococcus*, *Rothia* and *Ralstonia* encountered in lung cancer patients have also been found in the lungs of active and former smokers in the absence of disease [61,158], making it difficult to discern whether these alterations reflect cancer-specific processes or are influenced by prior exposure to tobacco smoke. Moreover, smoking-induced disruptions in the lung microenvironment may also allow the growth of potentially pro-carcinogenic microbes (e.g. producing carcinogenic compounds, interfering with pathways important for tumour formation/progression, promoting chronic inflammation and others). Smoking may therefore influence the microbial profiles detected in lung cancer patients either by promoting pro-tumourigenic microbial shifts or by confounding associations with cancer. In addition to smoking, other environmental factors may also contribute to the lung microbiome profiles identified in lung cancer patients. In this regard, household polycyclic aromatic hydrocarbon-rich coal (smoking coal) exposure and air pollution have both been found to affect lung microbiota diversity in lung cancer patients [119,126]. Furthermore, lung microbiota composition may be subject to the immunological state of the patient and treatment interventions. In fact, immunotherapy, chemotherapy, radiation and antibiotic use can significantly alter lung microbial populations and should also be taken into consideration when designing or interpreting experiments. Other variables that may influence lung microbiota profiles in lung cancer patients include age, sex, geographic location (as certain microbes display different geographic distribution patterns), comorbidities and clinicopathological characteristics (e.g. tumour histopathology, staging and others), factors that are increasingly being considered in studies in the field. Finally, certain technical factors may also affect results, including contamination issues, sampling approach and sequencing – data analysis methodology. For instance, it is frequently discussed that sampling passing through the upper respiratory tract (e.g. sputum, BALF), although less invasive, might lead to contamination and overrepresentation of oral taxa, especially considering the low-biomass profile of human lungs, whereas lung tissue obtained through thoracotomy circumnavigates oral contamination issues. The microbial profile of surgically excised lung tissue, on the other hand, may be affected by standard perioperative administration of antibiotics [159,160]. In addition to these issues, bronchoscopes commonly used to obtain BALF samples can only reach up to the fourth to fifth generation of bronchi [161], missing more peripheral sites. This might be an important factor, as a recent study comparing BALF samples obtained through bronchoscopy (upper lobe and bronchi) and lobectomy (intra-alveolar lavage) highlighted the existence of microbiota spatial variations across different lung sites [91]. Finally, although most studies have used the 16S rRNA sequencing approach (a method already prone to certain types of bias), newer analytical methods (metagenomics, metatranscriptomics) are increasingly taking centre stage, raising new data analysis standardization and interpretation issues [160]. Recognizing and taking into account these factors is thus critical when interpreting lung microbiome results in lung cancer, and tackling these (e.g. through patient stratification/application of multivariate statistical analyses) in future studies may result in clearer conclusions.

## Concluding remarks and suggestions for the future

Lung cancer currently represents a major healthcare challenge globally, and advances in early screening and targeted therapies are urgently needed to improve clinical outcomes. Despite the recent impetus in lung microbiome research suggesting an association between lung cancer and lung dysbiosis, significant obstacles preventing efficient translation remain. As with any nascent field, most efforts have so far concentrated on characterizing lung microbiomes (predominantly via 16S rRNA sequencing), while a multitude of biological and procedural factors can influence the results, further complicating our understanding. For instance, it is still difficult to confidently distinguish microbiome differences that actually relate to lung cancer processes from those influenced by biological confounders (e.g. inter-individual variability, effect of host genetic background, environmental exposures, treatment interventions) and study-specific experimental limitations (e.g. small sample size, grouping patients with different clinicopathological characteristics, 16S rRNA sequencing biases, sampling-analyses heterogeneities and contamination issues). Wider adoption of advanced sequencing techniques and culturomics, integration of multi-omic data and a focus on standardization and reproducibility in study designs and data analysis are likely necessary to obtain a more representative view of microbiome alterations in lung cancer. Considering the vast amounts of data that these technologies are poised to generate, increased application of artificial intelligence and machine learning may also be required for rapid gene annotation, functional gene prediction and inference of metabolic pathways, among others. In addition to studies focusing on the characterization and functional prediction of lung microbial communities, greater emphasis should be placed on directly assessing and interpreting the roles of microbes in lung physiology and cancer. Experimental validation of findings in a variety of different contexts/models is essential to determine whether microbial dysbiosis plays a causative role in lung cancer development and progression or if it is merely a byproduct of it, as well as in expanding our understanding of microbe–host interactions. In this regard, taxonomically distinct yet functionally relevant microbes (i.e. those sharing functional genes and pathways) should not be ruled out/overlooked. There is also a need for more studies systematically assessing the dynamics of interkingdom relationships (e.g. bacteria–fungi), as current evidence suggests that these likely play crucial roles in health and disease. Finally, making sense of the complex interplay among microbes, tumour cells, host microenvironment and external influences/environmental factors will benefit from systems biology and interdisciplinary expertise. Progress on these fronts, which is already underway, is expected to gradually illuminate the enigmatic role of microbes in lung cancer, paving new and more precise avenues for early lung cancer diagnosis and treatment.

## Abbreviations

BALF: bronchoalveolar lavage fluid
COPD: chronic obstructive pulmonary disease
CSC: cancer stem-like cell
EBV: Epstein–Barr virus
FEV1/FVC: forced expiratory volume in 1 s/forced vital capacity
GI: gastrointestinal
HPV: human papillomavirus
JCV: John Cunningham virus
LUAD: lung adenocarcinoma
LUSC: lung squamous cell carcinoma
MCPyV: Merkel cell polyomavirus
NSCLC: non-small cell lung carcinoma
OS: overall survival
ROS: reactive oxygen species
SCLC: small cell lung carcinoma
TIICs: tumour-infiltrating immune cells
